# The Book World of Medicine and Science

**Published:** 1907-05-18

**Authors:** 


### The Book World of Medicine and Science.

### STRAY NOTES.

**Indian Medicine.** Dr. Charles Moody relates his experiences among the American Indians in a chatty article which he contributes to a recent number of the *American Journal of Clinical Medicine.* Particularly interesting is his experience of the "medicine man." "As is often the case, if labour has not terminated so easily, the 'skiptiwat' is called in. He comes armed with all the panoply of his craft. I trust the reader will not accuse me of professional jealousy in dealing with this professional brother. He is an institution that no amount of civilisation has been able to eradicate. . . . . Dressed in his robes he enters the tent, shaking his elk's-tooth rattle. He seats himself before the woman, and proceeds to exorcise the evil spirit. This may be a pair of interlocked twins, a footling, a shoulder presentation, hydrocephalus, or any of the countless accidents of accouchement—it does not matter in the least to my savage *confrère*. He fills his lungs with smoke, and exhales it slowly upon the bare abdomen. Perhaps that is sufficient. At any rate he waits to see. If the woman is not delivered shortly he shakes his head, and tries another remedy. How like the civilised doctor. Another wait. No result following, he tries his last trump. This consists of drumming out the devil. All the while he is chanting in a way that would certainly frighten the wits out of any sensible devil. Sometimes the devil is obstinate, and does not avaunt. In that case the 'skiptiwat' is at the end of his resources and—lets the woman die." Dr. Moody relates how he gained his first firm hold upon the affections of his Indian patients by succeeding in a twin pregnancy where the skiptiwat had ignominiously failed, and tells some interesting experiences to show how, "no matter how thoroughly you Christianise an Indian, the ancestral idea of a future existence still remains with him."

**Students' Aids.** Examinations, so long as they foster the crammer and the cramming coach, will encourage condensed, desiccated cram books. The only justification for such manuals lies in the fact that many students find it worth while to go through their note-books the night before examination, and that such aids may take the place of the note-book. It is not altogether a valid justification. Personal note-taking, both at the bedside, in the out-patient department, and when reading, is far better than the reading of someone else's notes, no matter how good they may be, in record quick time before "going up." The series of "Students' Aid Manuals," isued by Messrs. Baillière, Tindall, and Cox, 8 Henrietta Street, W.C., is for those who have faith in such condensations, one of the best on the market. The volumes are cheap, excellently printed, and the information given in them is not altogether in tabloid form. The latest addition to the series is a handbook of "Diseases of Children," by John Caw, M.D., R.U.I., L.R.C.P.Edin., which is, indeed, a very good specimen of the best class of "cram book." There are many useful points in it, and the list of prescriptions at the end may be of service to the practitioner as well as to the student. The price is 4s. 6d. net.

**The Registers.** Every year the General Medical Council publishes two volumes, the "Medical Register" and the "Dentists' Register." The former is a bulky volume, retailed at half a guinea by Messrs. Spottiswoode and Co., Limited, 54 Gracechurch Street, E.C.; while the latter is much more slender, and costs 3s. 4d. This year's registers have been entirely revised and enlarged. Neither of them, of course, permits of much annual variety, but the "Medical Register" gives some interesting statistics. Thus we find that during 1906 1,197 names were added to the register, as against 1,270 in 1905, while 700 names were removed. Of these erasures 611 were due to deaths in the profession, and one only to "cessation of practice." The others were in consequence of action taken by the Council in accordance with Sections xiv., xviii., and xix. of the Medical Act. The number of registered practitioners whose names figure on the register is at present 39,620.

**The Derivation of Opsonin.** We take a good many things for granted, without making question of their accuracy. When Professor Wright gave to medical science a new word, and a new method of diagnosis, most people were content to accept the explanation that "Opsonin" was a derivative from a "well-known Greek verb." Now Dr. Moore points out, in the current number of the *Homœopathic Review,* that there is no such word, or verb, as "opsono" in Greek. "The name appropriately given by Professor Wright to these substances," he writes, "is from ὄψον, meaning boiled meats, anything eaten with bread to give it a relish, sauce, flavouring, or rich food. The word 'opson' (ὄψον) is a substantive derived from the verb *hepso,* meaning 'to boil,' when used of metals 'to smelt.' Whereas this verb is found no further back in old Greek literature than Pindar's Odes, the substantive ὄψον is used in the 'Iliad' and in the 'Odyssey' of Homer." There is a Latin derivative, *Opsono.*

**Cheap Reprints.** When there are so many excellently printed standard classics on the market, new "cheap editions" of old favourites are apt to be judged more strictly, perhaps even more harshly, than is justifiable. In many cases cheapness, at least in the production of books, is synonymous with inferiority, with bad paper and worse printing, with ill-read, badly spaced lines, and with poor, inartistic illustrations. To a certain extent the paper used in such reprints must be of a lower quality than that on which more expensive volumes are printed, but there is no reason why bad type and bad reading should combine to make the cheap edition unattractive. Messrs. T. Nelson and Sons, who were among the first to issue handy pocket volumes of our old favourites, have maintained a generally high standard in their cheap lines, and are at present issuing a series of shilling reprints, neatly bound in tooled cloth covers, of the works of Alexandre Dumas. "The Three Musketeers" is their latest production, and although it has been unduly cut down in the margins, it is a thoroughly acceptable book, handy in size, and not unworthy to figure among the more pretentious volumes which lie on the doctor's table.

---

Pathology: General and Special. A Manual for Students and Practitioners. By J. Stenhouse, M.A., B.Sc., M.B., and J. Ferguson, M.A., M.D. The Medical Epitome Series. (London: Hodder and Stoughton. 4s. net.)

This, the first number of a series designed to aid not only the student but "the general practitioner who might wish to refresh or supplement his knowledge to date," is an epitome of pathological knowledge. In some respects—for instance, the sections on teratology and tumours—it is a little too elaborate; in others (as in the too brief condensation of pancreatic lesions), it is scanty. Here and there one notices a statement which might well have been omitted, and there are numerous printer's errors. With these few failings, the little manual, which is judiciously illustrated, very ably fulfils its purpose, and may be cordially welcomed.

